# Full-length cDNA sequences from Rhesus monkey placenta tissue: analysis and utility for comparative mapping

**DOI:** 10.1186/1471-2164-11-427

**Published:** 2010-07-12

**Authors:** Dae-Soo Kim, Jae-Won Huh, Young-Hyun Kim, Sang-Je Park, Sang-Rae Lee, Kyu-Tae Chang

**Affiliations:** 1 National Primate Research Center, Korea Research Institute of Bioscience & Biotechnology, Ochang, Chungbuk 363-883, Republic of Korea; 2 Functional Genomics, University of Science and Technology, Daejeon 305-333, Republic of Korea

## Abstract

**Background:**

Rhesus monkeys (*Macaca mulatta*) are widely-used as experimental animals in biomedical research and are closely related to other laboratory macaques, such as cynomolgus monkeys (*Macaca fascicularis*), and to humans, sharing a last common ancestor from about 25 million years ago. Although rhesus monkeys have been studied extensively under field and laboratory conditions, research has been limited by the lack of genetic resources. The present study generated placenta full-length cDNA libraries, characterized the resulting expressed sequence tags, and described their utility for comparative mapping with human RefSeq mRNA transcripts.

**Results:**

From rhesus monkey placenta full-length cDNA libraries, 2000 full-length cDNA sequences were determined and 1835 rhesus placenta cDNA sequences longer than 100 bp were collected. These sequences were annotated based on homology to human genes. Homology search against human RefSeq mRNAs revealed that our collection included the sequences of 1462 putative rhesus monkey genes. Moreover, we identified 207 genes containing exon alterations in the coding region and the untranslated region of rhesus monkey transcripts, despite the highly conserved structure of the coding regions. Approximately 10% (187) of all full-length cDNA sequences did not represent any public human RefSeq mRNAs. Intriguingly, two rhesus monkey specific exons derived from the transposable elements of AluYRa2 (SINE family) and MER11B (LTR family) were also identified.

**Conclusion:**

The 1835 rhesus monkey placenta full-length cDNA sequences described here could expand genomic resources and information of rhesus monkeys. This increased genomic information will greatly contribute to the development of evolutionary biology and biomedical research.

## Background

The rhesus monkey (*Macaca mulatta*) is one of the species of Macaca, an Old World monkey. On the basis of DNA sequence comparison complemented by fossil evidence, the divergence of humans and Old World monkeys is estimated at about 25 million years ago [1]. The relationship between humans and rhesus monkeys is even more important because biomedical research has come to depend on these primates as experimental animal models [2]. Due to their genetic, physiologic, and metabolic similarity to humans, this species serves as an essential research tool in neuroscience, behavioral biology, reproductive physiology, neuroendocrinology, endocrinology, cardiovascular studies, pharmacology and many other areas [3-5].

The draft sequence of the rhesus monkey genome, which has an important evolutionary position, was published in 2007 [2]. The final challenge comes in the understanding of basic rhesus molecular biology through interpretation of the rhesus monkey genome. Transcriptome data could broaden the application of genome sequences. One of the most useful approaches obtaining large-scale sequence information is through the construction and sequencing of cDNA libraries [6]. These libraries represent a collection of genes that have been expressed as mRNA in a given cell or tissue, and are especially useful for obtaining sequence information on the coding regions of the genome [7]. Previous effort to catalogue the rhesus monkey transcriptome were based on expressed sequence tags (ESTs) used for the identification of genes, prediction of genes, and assessment of gene expression [8]. Also, a comparative analysis of mRNA sequences may provide clues to the genetic information that affects the different phenotypes [9]. However, the usefulness of EST clones is limited; because many EST clones lack the complete sequences of mRNAs, they cannot be used to reveal the primary structures of entire genes and encoded proteins [10]. ESTs are useful for making a catalog of expressed genes, but not for further study of gene function. Consequently, genome-scale collections of the full-length cDNAs of expressed genes become important for the analysis of the structure and function of genes [11]. In contrast to the great number of human full-length cDNA sequences in public databases such as the RefSeq mRNAs of the National Center for Biotechnology Information, only a small number of rhesus monkey mRNA sequences and ESTs have been deposited in public databases.

In this study, full-length-enriched cDNA libraries were constructed from rhesus monkey placenta using the oligo-capping method [12]. This method can efficiently identify apparent rhesus monkey homologs of human RefSeq mRNA sequences and collect the full insert sequence. Therefore, the full-length cDNA strategy was adopted to sequence and analyze a collection of 2000 cDNA sequences from placental cDNA library of a rhesus monkey. Full-length cDNA of the rhesus monkey will be beneficial for performing future genetic and biomedical studies.

## Results and Discussion

### Rhesus monkey cDNA library construction and sequencing

**Figure 1:**
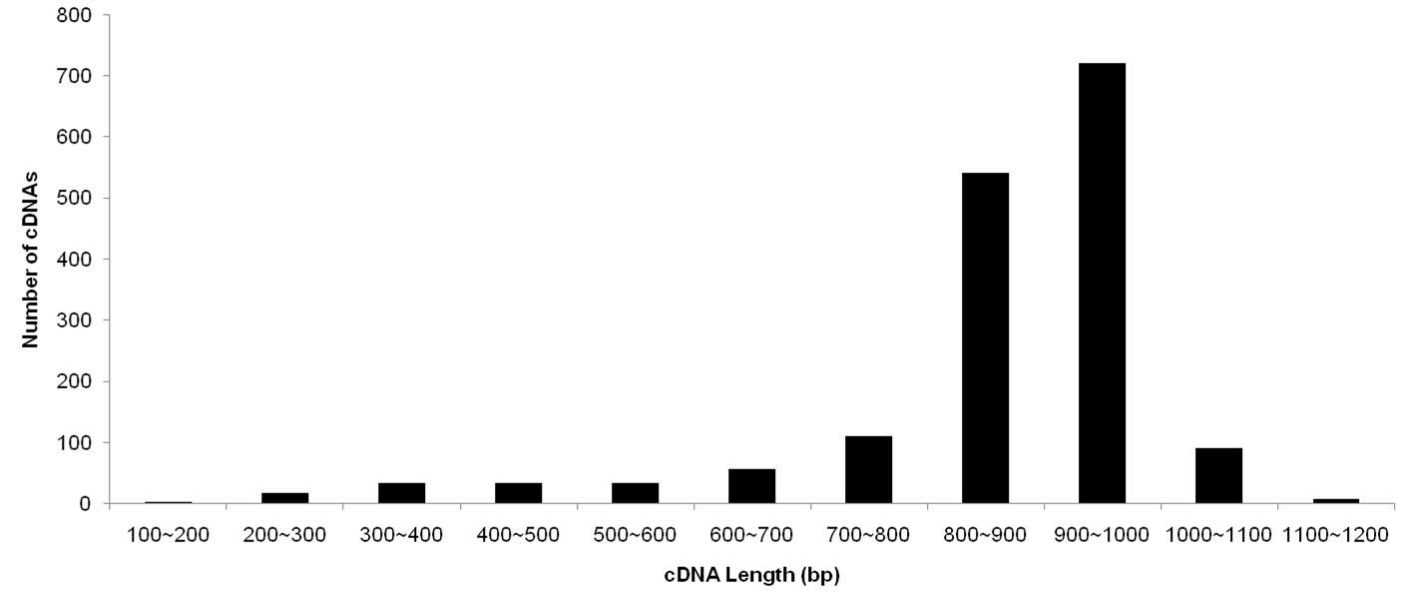
**Distribution of full-length placental cDNA lengths**.

Rhesus monkey placenta was harvested and used to generate a normalized, directional cDNA library. Around 2000 clones were randomly picked from the cDNA library and subjected to single-pass 5' sequencing using the cytomegalovirus primer located up-stream of the vector backbone. After trimming low-quality and vector sequences and removing contaminant host sequences, a total number of 1835 high-quality (quality score > 20) ESTs were obtained with a mean length of 858 bp. The length distributions of the ESTs are shown in Figure 1. cDNA length ranged from 170-1174 bp with an average of 858 bp. These average lengths were likely limited by sequencing technology. All cDNA sequences have been deposited in GenBank with continuous accession number of FS722297-FS724151.

### Gene identification and sequences analysis of known rhesus monkey cDNA sequences

The cDNA library derived from rhesus monkey placenta was constructed by the oligo-capping method. Two thousand sequences of rhesus monkey placental cDNA were annotated by the BLAST program. Since RefSeq sequences contain partially overlapped isoforms, non-redundant RefSeq sequences were constructed based on the Entrez Gene database. Consequently, 165 vector sequences that were included in the raw sequence data were eliminated. To classify these rhesus monkey cDNAs and find their human homologues, BLAST searches were performed to human RefSeq databases. Of the 1835 cDNA sequences, 1648 were homologous to the human RefSeq gene set and were clustered into 1462 types of genes, indicating that the cDNA sequences would cover approximately 4.8% of the known human genes (Table 1). Using the protein coding gene set of the rhesus monkey cDNA as defined by human RefSeq mRNA database, the frequency of mapping in 5'untranslated repeat (UTR), 5'UTR_CDS, CDS, CDS_3'UTR, 3'UTR, and 5'UTR-CDS-3'UTR was investigated at various distances from genes. Of the annotated 1648 cDNA transcripts, only about 86% of these (1590 transcripts) were present within the known gene regions: 20 mapping in 5' UTR, 143 in 5'UTR_CDS, 142 in CDS, 549 in CDS_3'UTR, 532 in 3UTR, and 204 in 5'UTR-CDS-3'UTR (Table 1). Of those that matched human RefSeq mRNAs, 204 cDNA sequences contained the full coding region with 5'UTR and 3'UTR sequences. Although the oligo-capping cDNA library construction method was aimed to generate full-length cDNA sequences, the results indicated that only 367 transcripts were derived by 5'UTR sequences. This relative low percentage of full-length inserts was mainly due to the fact that the method used was not optimized to generate a full-insert cDNA library.

**Table 1:** Annotation of rhesus monkey placenta cDNA sequences using human RefSeq mRNA

Index	Human RefSeq mRNA		
	
Mapping Region	cDNA	RefSeq	Percent (%)
5UTR	20	19	1.3
5UTR_CDS	143	134	9.2
CDS	142	137	9.4
CDS_3UTR	549	499	34.1
3UTR	532	443	30.3
5UTR-CDS-3UTR	204	181	12.4
NR and XR	58	49	3.4
Total	1648	1462	100.0

Another study reported the initial sequencing and comparative analysis of rhesus monkey cDNA sequences from 11 tissues. These 48,642 sequence data from three different macaque species represented an initial sampling of the putative rhesus orthologs for 6216 human genes, and the researchers focused on the genetic divergence between the human and non-human primate [7]. Because their cDNA libraries were constructed from the poly (dT)-primed cDNA, their method for Uni-ZAP cDNA library construction could not aim at the full-length cDNA sequences. However, the present oligo-capping method with normalization could capture non-redundant full-length mRNA sequences. To declare the differences between the present and previous [7] data, the prior results were reanalyzed. Although a total of 48,642 sequence reads from 11 rhesus monkey tissues had been sequenced, the present reanalysis retrieved and analyzed placenta tissue data sets of 12,033 sequences. Of these sequences, 8340 cDNA sequences corresponding to 2390 human RefSeq genes (7.8% of all human RefSeq genes) were collected (Additional file 1). Intriguingly, only 284 genes overlapped with the previous analysis. Although the main target of placenta tissues is the same, their results showed quite different data sets. These different results reflect the gap in knowledge in the identification and analysis of rhesus monkey genes.

### Similarity analysis between human and rhesus monkey cDNA sequences

**Figure 2:**
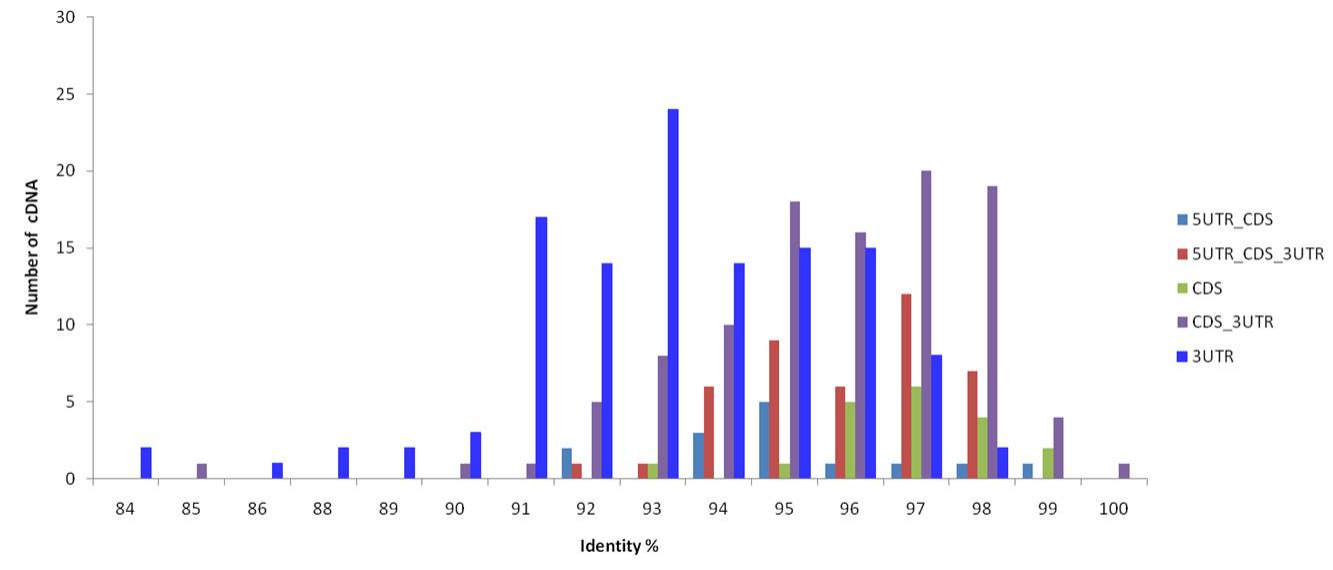
**Distribution of sequence identity of full-length cDNA of rhesus monkey placenta**.

From the 1835 sequences, consensus sequences could be constructed to 138 rhesus monkey genes by aligning with at least two sequences of individual genes and the nucleotide sequence identity between humans and rhesus monkey. Sequence identities were calculated between 298 rhesus monkey consensus sequences and the corresponding human RefSeq mRNA sequences. These 298 consensus cDNA sequences of 138 rhesus monkey genes aligned with 5'UTR_CDS_3'UTR, 5'UTR-CDS, CDS, CDS_3'UTR and 3'UTR regions of human RefSeq mRNAs (Figure 2). The average sequence identity of the 5'UTR_CDS in 14 cDNAs was 95.1%, 5'UTR_CDS_3'UTR in 42 cDNAs was 95.9%, CDS in 19 cDNAs was 96.8%, CDS_3'UTR in 104 cDNAs was 95.7%, and 3'UTR in 119 was 93.3%. The patterns of divergence were different from the 5'UTRs, CDSs, and 3'UTRs. The sequence divergence in the 5'UTRs and CDS region was significantly lower than that of 3'UTR sequences. These results coincided with a previous study indicating the sequence identity between human and rhesus monkey cDNA was 97.79% in coding sequence and 95.10% in 3'UTRs [7]. Upstream and coding regions of functional genes are very important [13]. Thus, these regions are evolutionary well-conserved in comparison with 3' UTR regions. Specifically, sequence substitution in the CDS region could disrupt the important functional domain or coding frames. Moreover, this substitution in the 5'UTR region also could interrupt the binding interaction between regulatory binding sites and regulatory elements.

Use of the present oligo-capping method enabled the construction of full-length-enriched cDNA libraries from rhesus monkey placenta tissue. Although various genomics projects have focused on sequencing of the genome or ESTs, full-length cDNA sequences are uniquely informative resources for accurately predicting the full spectrum of the transcriptome in specific species [14].

### Analysis of unknown transcripts

**Figure 3:**
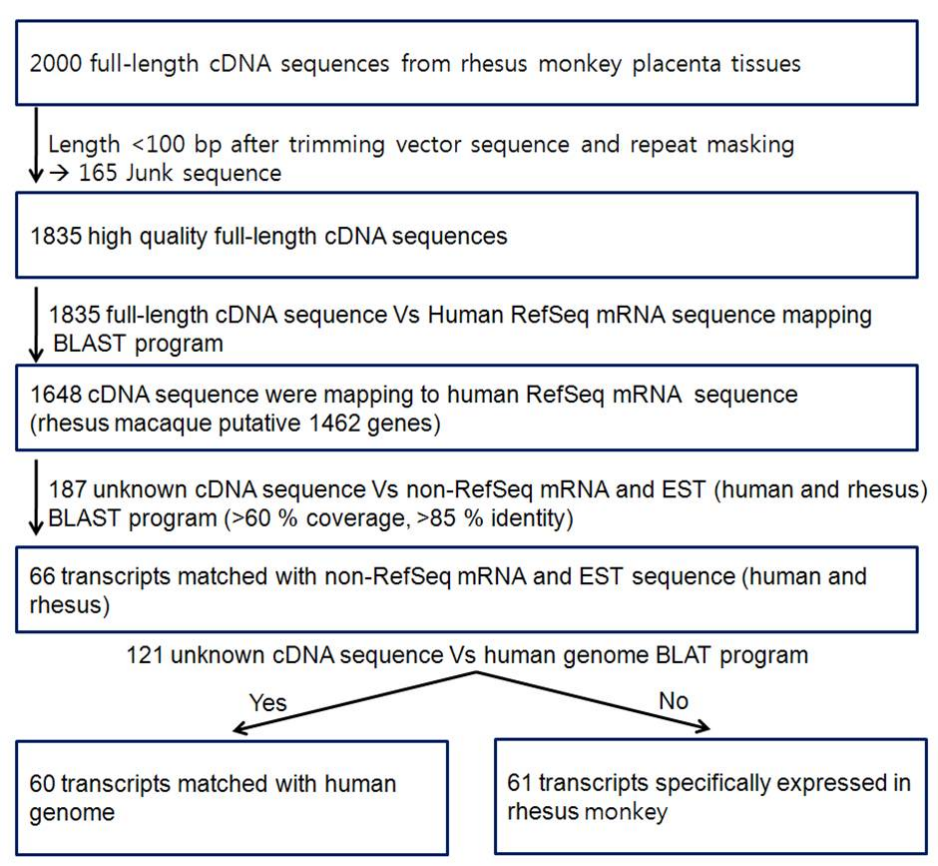
**A flow chart showing the overall procedure for searching for putative rhesus monkey genes**.

Of the 1835 placenta cDNA sequences, 187 sequences were not homologous to the human RefSeq mRNA sequences (Figure 3). Although 66 transcripts are not annotated with human RefSeq mRNA sequences, their sequences matched with the non-RefSeq mRNA (human and rhesus) and ESTs (human and rhesus) sequences in the database. Finally, 121 transcripts were designated as unknown transcripts.

To characterize the 121 unknown transcripts, two individual strategies were established. One is the human genome aligning method with open reading frame (ORF) coding method (60 transcripts). The other uses only the ORF coding method (61 transcripts). Among the 121 transcripts, 60 transcripts matched with the human genome using BLAST (Table 2). Remarkably, these 60 transcripts also contained ORFs with lengths ranging from 44-198 amino acids (data not shown). These unknown transcripts were identified in known gene regions or intergenic region. Furthermore, their matching patterns in human genome were similar with splicing patterns. These matching regions were designated as "block". Although detailed characterization and experimental validation of these 60 transcripts was not done, they could be valuable sources for the novel gene candidates.

**Table 2:** Mapping results of the 60 cDNAs to the human genome.

GenBank Acc	Length (bp)	Matched length (bp)	Query Start	Query End	Chr	# of Blocks	Aligned region^a^
FS722584	827	765	62	827	chr11	6	NAT10 (Intron,Exon)
FS723598	889	648	69	717	chr1	8	Intergenic
FS723503	850	598	252	850	chr2	3	Intergenic
FS723108	829	770	59	829	chr11	7	Intergenic
FS723805	823	627	57	684	chr5	2	PPP2R2B (Intron)
FS722908	835	774	61	835	chr3	6	Intergenic
FS723536	865	776	78	854	chr7	4	Intergenic
FS723202	936	758	155	913	chr5	6	Intergenic
FS722459	525	462	63	525	chr7	2	LOC493754 (Intron)
FS722694	849	795	54	849	chr14	7	Intergenic
FS723758	861	807	54	861	chr3	7	DVL3 (Intron,Exon), AP2M1 (Intron)
FS723877	964	900	60	960	chr7	5	MKLN1 (Intron)
FS723612	933	876	56	932	chr15	3	Intergenic
FS722689	942	720	222	942	chr1	8	FLEKHA6 (Intron)
FS723970	222	188	34	222	chr11	2	TIMM10 (Intron,Exon)
FS723532	1000	645	57	702	chr16	7	GABARAPL2 (Exon, Intergenic)
FS723424	943	546	59	605	chr20	2	ITCH (Intron, Exon)
FS723884	955	678	57	735	chr17	7	SCN4A (Intron)
FS723781	961	900	61	961	chrX	10	Intergenic
FS723422	972	752	61	813	chr15	10	Intergenic
FS724052	843	752	88	840	chr3	6	ITPR1 (Intron)
FS722839	934	868	48	916	chr5	3	IL4 (Intron)
FS723579	853	735	118	853	chr9	7	Intergenic
FS722478	938	768	57	825	chr22	5	Intergenic
FS723804	867	800	62	862	chr6	2	PKHD1 (Intron)
FS723444	967	795	172	967	chr7	5	Intergenic
FS723329	921	859	62	921	chr11	12	Intergenic
FS723221	954	560	153	713	chr1	3	Intergenic
FS723414	1013	946	48	994	chr2	7	Intergenic
FS723140	816	701	115	816	chr2	5	Intergenic
FS722423	886	822	64	886	chr2	4	MYEOV2 (Intron)
FS722597	913	648	118	766	chr4	6	Intergenic
FS723463	939	632	61	693	chr21	5	Intergenic
FS723411	241	184	56	240	chr7	3	Intergenic
FS723556	891	833	57	890	chr16	7	Intergenic
FS723572	771	706	65	771	chr6	4	Intergenic
FS722560	943	880	61	941	chr10	6	NPR1 (Intron)
FS723477	1024	557	106	663	chr2	5	Intergenic
FS723929	957	727	230	957	chr10	5	Intergenic
FS723671	174	93	62	155	chr17	2	ORMDL3 (Intron)
FS724061	855	477	29	506	chr2	6	Intergenic
FS722691	864	637	39	676	chr6	4	Intergenic
FS723333	941	847	62	909	chrX	5	Intergenic
FS722546	1021	933	63	996	chr6	11	GMPR (Intron)
FS723142	886	762	76	838	chr20	4	SLC13A3 (Intron)
FS723357	756	691	65	756	chr6	6	BET3L (Intron)
FS722638	961	894	41	935	chr9	6	Intergenic
FS723442	887	654	41	695	chr1	4	Intergenic
FS723880	810	695	46	741	chr21	9	HUNK (Intron,Intergenic)
FS723438	1012	556	422	978	chr9	8	Intergenic
FS723883	624	393	181	574	chr10	3	Intergenic
FS723617	1034	966	48	1014	chr9	12	VLDLR (Intron)
FS723491	859	575	55	630	chr17	2	Intergenic
FS723767	885	636	63	699	chr9	2	AUH (Exon, Intron)
FS723901	822	591	137	728	chr2	6	C2orf67 (Intron)
FS723913	891	791	60	851	chr1	8	Intergenic
FS723200	832	750	66	816	chr5	6	Intergenic
FS723126	854	799	55	854	chr9	5	Intergenic
FS723264	1039	976	56	1032	chr1	5	PLXNA2 (Intron)
FS724105	746	379	136	515	chr3	3	Intergenic

^a^, The aligned regions were determined by comparing the BLAST results with the refGene database.

Among the 121 transcripts, 61 were analyzed only using ORF coding. Because these 61 transcripts did not share significant similarities with any human genome, they were anticipated to be rhesus monkey specific transcripts. However, due to the limitation of rhesus monkey genome availability, this analysis could not be done. These 61 unknown transcripts clearly contain ORF regions that average 106 amino acids in length (data not shown). These results should also prove interesting for the identification of lineage specific rhesus monkey transcripts.

The results of species specific transcripts indicated in the present analysis are consistent with recent comparative analysis between human and chimpanzee [15]. These species specific transcripts could be informative clues for the explanation of different characters. The rhesus monkey is one of the most widely-used and valuable biomedical animal models for the investigation of numerous human diseases. This information would provide a better understanding of the genetic information of rhesus monkey species.

### INDEL analysis

**Figure 4:**
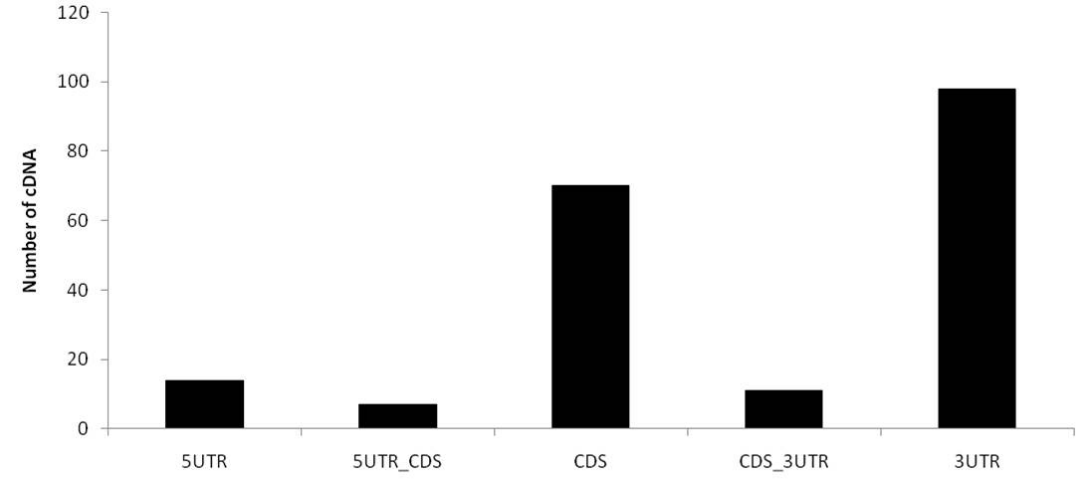
**INDEL sequences of placenta expressed transcripts between human and rhesus monkey**.

INDEL sequences of placenta expressed transcripts were analyzed between human and rhesus monkey. If a sequence was aligned in more than one place in a genome, only the high-scoring sequence pair alignments was kept to ensure that a sequence mapped to a single locus. We selected human and rhesus monkey gene regions bearing nucleotide INDELs when aligned to the genome. A total of 214 transcripts showed different transcript structures. Next, the frequency of INDELs was investigated in 5'UTR, 5'UTR_CDS, CDS, CDS_3'UTR, and 3'UTRs in putative rhesus monkey genes. Of the 214 INDELs regions, 200 were located in functional gene regions including 14 in 5'UTR, seven in 5'UTR_CDS, 70 in CDS, 11 CDS_3'UTR, and 98 in 3'UTR regions (Figure 4). Moreover, the INDEL density in the 3'UTRs was significantly lower than the 5'UTR regions. INDELs could disrupt important motifs in regulatory regions and also alter the spacing between regulatory binding sites. The present analysis revealed low INDEL density in the 5'UTR region and high INDEL density in the 3'UTR region and, especially, the CDS region. These results may be explained by selection acting against INDELs in specific regions.

**Figure 5:**
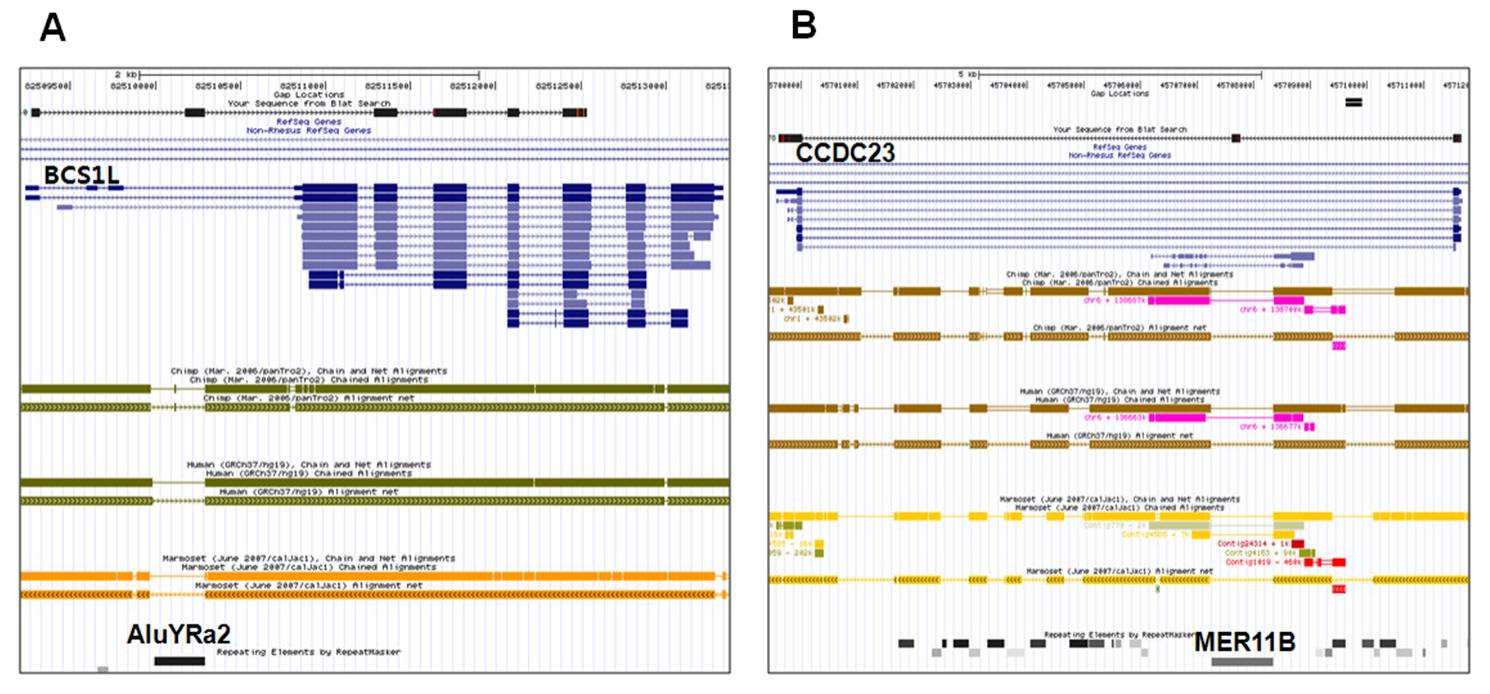
**Rhesus monkey specific exon in BCS1L and CCDC23 genes**. Part of the annotated rhesus genome available in the UCSC Genome Browser Database is shown. The dark black region is a sequencing gap, which spans exons 1 and 2 of human BCS1L. The bottom track indicates the human, chimpanzee, and marmoset regions that correspond to the orthologous rhesus chromosome. Symbols: thick, aligned; thin, unaligned due to either deletion in the chromosome (human, chimpanzee, marmoset) or sequencing gap. Arrows indicate transcription orientation. Blue blocks indicate exons. (B) The dark black region is a sequencing gap, which spans exons 1 and 2 of human CCDC23.

Presently, a screening procedure was developed to identify putative rhesus monkey specific exons with transposable elements in alternative splicing events. Exons were analyzed using rhesus monkey placenta cDNA sequences. The 214 candidates were manually inspected to narrow them to two highly plausible cases: exons that exhibited alternative splicing patterns in the rhesus monkey but not in humans. Moreover, some alternative splicing events were rhesus monkey specific: the rhesus monkey exon of the BCS1L gene was derived from rhesus monkey specific AluYRa2 elements (Figure 5a). AluYRa2 is commonly considered to be a rhesus monkey specific Alu element [16]. In addition, the rhesus monkey specific exon of CCDC23 was derived from MER11B elements (Figure 5b). Intriguingly, two rhesus monkey specific exons were derived from the transposable elements of AluYRa2 (SINE family) and MER11B (LTR family). These two transposable elements are abundant in different genomic regions including intergenic regions and intron regions. The consensus TE element, specifically Alu elements, carries multiple sites that are similar, but not identical, to the real splice site [17]. Therefore, these two elements could be frequently exonized as primate-specific exons and could thus be used to identify the BCS1L and CCDC23 genes showing rhesus monkey specific INDELs.

The present study demonstrated the evolutionary occurrence of species-specific exons by alternative splicing of transposable elements in the gene during evolution. Although an absolute determination of species specificity will require complete rhesus monkey genome sequencing or experimental validation, preliminary computational analysis could be done to test the presence or absence of these sequences in the human and other primate genomes. Several studies have characterized the series of mutations needed occur within a transposable element in order to generate a new exon [18]. For example, it was described in the human RNA editing enzyme ADAR2, where exon 8 is a new *Alu*-derived primate-specific exon that is alternatively spliced in high inclusion levels [19] in a tissue-regulated manner. The new exon is inserted in the catalytic domain of ADAR2 and, while the exon-containing variant has the same substrate specificity as the original one, it has an altered catalytic activity [20]. Moreover, 62% of new exons in human are associated with primate-specific *Alu *retroposons, and 28% of new exons in rodents are derived from rodent-specific SINEs [21]. Therefore, it seems that, in primates, the transposable elements exonization mechanism is being used as a major source for acetated, lineage specific evolution, and is perhaps a key driving force to eventual speciation.

## Conclusions

In the present study, a cDNA library was constructed using an oligo-capping method from the placenta of the rhesus monkey, and approximately 2,000 randomly picked normalized clones were sequenced. The 1835 rhesus monkey placenta full-length cDNA sequences described here significantly expands the molecular resources available for the genus. Further analysis revealed 121 transcripts in rhesus monkey cDNA, in which 61 did not share significant similarities with any human genome. Such sequence information was used in comparative analysis to identify novel genes specifically expressed in rhesus monkey. Moreover, some alternative splicing events are rhesus monkey specific: rhesus monkey exons of BCS1L and CCDC23 genes were derived from rhesus monkey specific AluYRa2 and MER11B elements, respectively. Increasing the genomic resources and information of rhesus monkeys will greatly contribute to the development of evolutionary biology and biomedical sciences. The construction of a large scale collection of full-length cDNA sequence from rhesus monkey placenta tissue and homology searches in databases would facilitate the discovery of novel genes.

## Methods

### Rhesus monkey placenta tissue

The placenta sample was collected from an 8-year-old female rhesus monkey of Chinese origin during cesarean delivery. All animal housing and experiments were performed in accordance with Korea Research Institute of Bioscience and Biotechnology (KRIBB)Institutional Animal Care and Use Committee Guidelines (Accepted No. KRIBB-AEC-09017).

### Construction of full-length enriched normalized cDNA library

Collected placenta tissue was immediately frozen in liquid nitrogen and used for RNA extraction. Modified oligo-capped cDNA libraries were constructed according to a previously described method [12]. After the construction of the full-length enriched cDNA library, normalization steps were carried out as previously described [22]. During all steps for full-length cDNA library construction and normalization, a total of five steps were checked (RNA isolation step, first cDNA synthesis step, second cDNA synthesis step, transformation step, and normalization step). All these procedures were conducted by CoreBioSystem (Korea).

### Sequencing of cDNA clones

The cDNA clones were sequenced with ABI 3730 automated sequencers. Sequencing of size-selected 2000 clones was determined by a commercial sequencing company (Cosmo Genetech). For the sequencing of 5' region of full-length cDNA sequences, a cytomegalovirus primer (5' CGC AAA TGG GCG GTA GGC GTG 3') was used. The rhesus monkey cDNA sequences were deposited in the public DNA databases [DDBH/EMBL/GenBank: FS722297-FS724151].

### Data set

Human genome build 37.1, NCBI RefSeq mRNA (12 March 2009), and the June 2006 NCBI rhesus build 1 genome were used as the reference databases for all analyses. Transposable elements in the rhesus monkey genome sequences were identified by RepeatMasker http://repeatmasker.genome.washington.edu, and transposable element consensus sequences were identified by Repbase Update [23].

### Human-rhesus monkey cDNA sequence alignment

A total of 2000 cDNA sequences were selected. cDNA sequences were first base-called using a modified version of the phred algorithm [24] and then screened for cloning vector, lambda-phage, and *Escherichia coli *contamination using the cross_match program [25]. Sequences exhibiting multiple cloning sites or any contamination with lambda-phage or *E. coli *sequence were excluded from further analysis [26]. The result was the generation of a clean, high-quality EST sequence set. After trimming low-quality and vector sequences and removing contaminant host sequences, a total number of 1835 high-quality ESTs were obtained with a mean length of 858 bp. The BLAST program was used to align each rhesus monkey cDNA sequence with the human RefSeq mRNA sequence. Whenever BLAST failed to align rhesus monkey cDNA sequence with human RefSeq mRNA sequence, it was compared to the rhesus monkey genome database using the BLASTN program [27]. The rhesus monkey sequences that matched the sequences of human RefSeq mRNAs were defined as the sequences of putative rhesus monkey genes. The rhesus monkey cDNA sequences were then mapped on the draft genome sequence of the rhesus monkey.

## Abbreviations

CDS: coding sequence; EST: expressed sequence tag; ORF: open reading frame; UTR: untranslated region;

## Authors' contributions

DS Kim analyzed contents of this paper and drafted the manuscript. JW Huh designed the experiment on the whole study and revised the manuscript. YH Kim and SJ Park performed the experiments and analyzed the data. KT Chang participated in its analysis and provided essential direction.

All authors read and approved the final manuscript.

## Supplementary Material

Additional file 1 **Rhesus monkey placenta cDNA sequences mapping with human RefSeq mRNA transcripts**. Click here for file
